# Association of alkalization therapy with long-term survival in stage IV or recurrent colorectal cancer: interaction with RAS mutation status

**DOI:** 10.3389/fonc.2026.1867616

**Published:** 2026-08-07

**Authors:** Kazuyuki Suzuki, Shion Kachi, Reo Hamaguchi, Hiromasa Morikawa, Hiromi Wada

**Affiliations:** 1 Department of Informatics, The University of Electro-Communications, Chofu, Tokyo, Japan; 2 Japanese Society on Inflammation and Metabolism in Cancer, Kyoto, Japan

**Keywords:** alkalization therapy, colorectal cancer, liver metastasis, overall survival, RAS mutation, stage IV colorectal cancer, tumor microenvironment, urinary pH

## Abstract

**Background:**

Stage IV or recurrent colorectal cancer remains difficult to treat, especially in patients with RAS-mutant tumors, for whom anti-EGFR antibodies are not effective. Increasing evidence indicates that acidification of the tumor microenvironment promotes invasion, metastasis, therapeutic resistance, and immune suppression. Alkalization therapy has therefore attracted interest as a supportive strategy to improve systemic acid-base and metabolic conditions.

**Methods:**

We retrospectively analyzed patients with stage IV or recurrent colorectal cancer who first visited Karasuma Wada Clinic between 2014 and 2019 and underwent alkalization therapy with at least three urinary pH measurements. Two patients with BRAF-mutant tumors were excluded, leaving 129 patients for the primary analysis. Urinary pH was summarized using the 25% chronologically trimmed mean urinary pH. Overall survival (OS) was the primary endpoint. Among 80 patients with known RAS mutation status, a Cox proportional hazards model including interaction terms for urinary pH × RAS mutation status and urinary pH × liver metastasis was used as the primary analysis, followed by Kaplan-Meier analyses guided by the interaction structure.

**Results:**

In the overall cohort, the 5-year OS rate was 0.212 (95% CI, 0.113-0.362). Among the 80 patients with known RAS status, the interaction between urinary pH and RAS mutation status was significant (HR, 0.22; 95% CI, 0.06-0.78; likelihood ratio test, p = 0.0199), indicating that the prognostic impact of urinary pH differed according to molecular background. The 5-year OS rates were 0.250 in the RAS wild-type group and 0.130 in the RAS mutation-positive group. Within the RAS mutation-positive subgroup, patients with urinary pH ≥7.0 had significantly better OS than those with urinary pH <7.0 (Gehan-Breslow-Wilcoxon test, p = 0.013). Among patients with both RAS mutation-positive disease and liver metastases, urinary pH ≥7.0 was likewise associated with significantly longer OS (log-rank p = 0.0141).

**Conclusions:**

Maintenance of urinary pH ≥7.0 under alkalization therapy was associated with longer survival in stage IV or recurrent colorectal cancer, particularly in clinically unfavorable subgroups defined by RAS mutation and liver metastasis. These findings support prospective evaluation of alkalization-oriented intervention as a complementary therapeutic strategy.

## 1 Introduction

Colorectal cancer remains one of the leading causes of cancer incidence and mortality worldwide, and stage IV or recurrent metastatic colorectal cancer continues to represent a major therapeutic challenge despite advances in systemic chemotherapy and molecularly targeted agents (1–3). In particular, RAS mutation status is directly linked to treatment selection, because anti-EGFR antibodies such as cetuximab and panitumumab are effective only in RAS wild-type disease and are fundamentally ineffective in RAS-mutant tumors (2–4). Therefore, patients with RAS mutation-positive metastatic colorectal cancer often have fewer therapeutic options and poorer prognosis, underscoring the need for complementary therapeutic strategies that act through mechanisms distinct from direct oncogene targeting.

At the same time, advances in cancer metabolism and tumor microenvironment (TME) research have revealed that extracellular acidification around tumors is closely linked to invasion, metastasis, drug resistance, and immune suppression. Cancer cells enhance aerobic glycolysis through the Warburg effect, produce large amounts of lactate and protons, and export them through monocarboxylate transporters (MCTs). In addition, pH-regulatory systems such as the Na^+^/H^+^ exchanger (NHE1) and carbonic anhydrase IX (CAIX) are rewired, allowing tumor cells to maintain a relatively alkaline intracellular pH while lowering extracellular pH (pHe) (5–12). This reversed pH gradient is thought to promote tumor survival advantages through mechanisms such as matrix degradation and invasion via increased protease activity, suppression of T-cell and NK-cell function, and reduced uptake of cytotoxic agents (6, 13–16).

Against this background, “pH-targeted therapy” aimed at alleviating TME acidification has been investigated at the preclinical level. Buffering strategies using bicarbonate or citrate salts have been reported to increase extracellular tumor pH and to suppress metastasis or improve therapeutic sensitivity (10, 17, 18). In addition, neutralization of tumor acidity has been shown to restore antitumor immunity and to enhance the efficacy of immunotherapy (14, 16, 17). Based on these basic and preclinical findings, we and others have clinically introduced alkalization therapy in patients with advanced non-small cell lung cancer and stage IV pancreatic cancer and reported associations between urinary pH, defined by the 25% chronologically trimmed mean urinary pH, and long-term outcomes (19–23). In those studies, patients who maintained higher urinary pH under alkalization therapy showed prolonged overall survival and a characteristic long-tail survival pattern (19–21, 23). However, as emphasized in the most recent narrative review, urinary pH should not be interpreted as a direct measure of local extracellular tumor pH; rather, it should be regarded as a clinically available indirect supportive marker of systemic acid-base and metabolic conditions (23). Nevertheless, these findings suggest that correction of the acidic tumor microenvironment may contribute to long-term survival across tumor types.

To our knowledge, no clinical study has systematically examined the prognostic relevance of alkalization therapy specifically in patients with stage IV or recurrent colorectal cancer. Clinical evidence remains scarce regarding the extent to which pH control of the tumor microenvironment may affect survival in subgroups with strong adverse prognostic factors, such as RAS-mutant disease and liver metastasis. Therefore, in the present study, we retrospectively investigated the association between urinary pH under alkalization therapy and long-term prognosis in patients with stage IV or recurrent colorectal cancer who first visited Karasuma Wada Clinic. In particular, through stratified analyses according to RAS mutation status and liver metastasis, we explored whether alkalization therapy might partly overcome poor prognosis even in subgroups for which standard molecularly targeted treatment is less effective.

## 2 Materials and methods

### 2.1 Study design

This study was a single-center retrospective observational cohort study of patients with stage IV or recurrent colorectal cancer treated at Karasuma Wada Clinic. The study was based on retrospective analysis of medical records and laboratory data. The study was conducted in accordance with the Declaration of Helsinki, approved by the Institutional Review Board of the Labor and Safety Office, The University of Electro-Communications (approval number H24052), and registered with the UMIN Clinical Trials Registry (UMIN000057017; date of registration: 14 February 2025). Because this was a retrospective observational study using existing clinical data, participation was handled through an opt-out procedure in accordance with institutional requirements.

### 2.2 Patients

We identified patients with colorectal adenocarcinoma who first visited Karasuma Wada Clinic between January 2014 and December 2019 and who either had stage IV disease at initial presentation or developed distant metastasis or local recurrence after prior curative-intent surgery. Patients were eligible for inclusion if they met the following criteria: (1) urinary pH had been measured at least three times during outpatient visits after the first visit to Karasuma Wada Clinic, and (2) their general condition was considered suitable for alkalization therapy. Two patients with BRAF-mutant tumors were excluded because the number of such cases was too small for meaningful evaluation, leaving a final study population of 129 patients.

RAS/BRAF mutation status was collected primarily from prior pathology-based molecular testing documented in referral letters and medical records. Patients were categorized as RAS wild-type, RAS mutation-positive, BRAF mutation-positive (excluded), or unknown. Baseline information recorded at the first visit included age, sex, postoperative recurrence status, and metastatic sites, including liver, lung, lymph nodes, peritoneum, and bone. Liver metastasis was classified as absent, liver-only, or liver plus other organs.

All patients received standard systemic treatment, including chemotherapy and molecularly targeted therapy (such as anti-EGFR or anti-VEGF antibodies), either at referring hospitals or at collaborating core hospitals linked to Karasuma Wada Clinic. In the present study, the primary focus was not on detailed comparison of treatment regimens, but rather on the relationship between urinary pH and survival.

### 2.3 Alkalization therapy

Alkalization therapy was defined as a treatment strategy based on an alkaline diet, aimed at alleviating tumor microenvironmental acidification and optimizing systemic acid-base and metabolic conditions, as reflected in part by urinary pH, with the optional addition of oral sodium bicarbonate, citric acid, or both according to each patient’s clinical condition (23).

An alkaline diet was defined as a diet rich in vegetables and fruits and reduced in meat and dairy products, with the aim of lowering dietary acid load and supporting systemic acid-base and metabolic conditions. Urinary pH was used as a practical clinical marker to monitor the response to alkalization therapy.

According to each patient’s urinary pH, general condition, disease status, and clinical judgment, oral sodium bicarbonate, citric acid, or both were additionally prescribed when considered necessary. Therefore, the components of alkalization therapy differed among patients and included dietary intervention alone, dietary intervention plus sodium bicarbonate, dietary intervention plus citric acid, and dietary intervention plus both sodium bicarbonate and citric acid.

Patients were asked to record their daily meals for at least the first 4 weeks after starting the alkaline diet, when feasible, to assess whether the dietary intervention was being followed appropriately. Based on these dietary records, physicians or nurses provided individualized dietary advice. At each visit, patients also received guidance regarding the alkaline diet, and adherence to the dietary intervention was assessed through interviews, dietary records when available, and serial urinary pH measurements.

At each outpatient visit, spot urine samples were collected and urinary pH was measured using either dipsticks or a pH electrode. A urinary pH of ≥7.0 was used as one practical target during alkalization therapy, and lifestyle guidance and medication were adjusted individually according to the longitudinal urinary pH profile. Urinary pH has been reported to reflect changes in dietary acid load and proton excretion (24).

For quantitative evaluation of urinary pH, we used the 25% chronologically trimmed mean urinary pH. This was defined by arranging serial urinary pH measurements in chronological order, excluding the earliest 25% and the latest 25% of measurements, and calculating the mean of the remaining middle 50%. This method was used to reduce the influence of short-term fluctuations and outliers while capturing the long-term alkalization status of each patient.

### 2.4 Data collection

The following variables were retrospectively extracted from the medical records:

• patient background: age, sex, disease status at first visit (*de novo* stage IV vs postoperative recurrence), primary tumor site, and prior treatment history;
• tumor-related factors: RAS/BRAF mutation status, metastatic sites (liver, lung, lymph nodes, peritoneum, bone, etc.), and liver metastasis pattern;
• treatment-related factors: systemic chemotherapy regimens before and after the first visit, and the use of targeted agents such as anti-EGFR and anti-VEGF antibodies;
• alkalization-related factors: longitudinal urinary pH values and the 25% chronologically trimmed mean urinary pH;
• outcome: date of death or date of last follow-up.

The primary outcome was overall survival (OS), defined as the interval in days from the first visit to Karasuma Wada Clinic until death. Patients who were alive were censored at the last follow-up date.

### 2.5 Statistical analysis

Continuous variables are presented as medians with interquartile ranges, and categorical variables as frequencies and percentages.

The main analysis was designed to test whether the effect of urinary pH on OS was uniform across the cohort or modified by clinically relevant biological background. Accordingly, a Cox proportional hazards model was fitted with interaction terms for urinary pH × RAS mutation status and urinary pH × liver metastasis. In the primary model, urinary pH was treated as a continuous variable using the 25% chronologically trimmed mean urinary pH, in order to preserve information and reduce dependence on an arbitrary cutoff. For clinical interpretability, a sensitivity analysis was also performed using dichotomized urinary pH groups defined as ≥7.0 versus <7.0.

When an interaction term was significant, the main effects of urinary pH and RAS mutation status were not interpreted in isolation. Instead, the effect of urinary pH was presented through stratified estimation according to RAS status, using either stratified Cox modeling or conditional interpretation of the interaction model. The proportional hazards assumption was checked, including after stratification, and results were interpreted cautiously if any meaningful deviation was suspected. To minimize overfitting, the number of covariates entered into multivariable models was limited in consideration of the number of events.

On the basis of the structure suggested by the interaction model, OS was then visualized using the Kaplan–Meier method, and survival curves were compared using the log-rank test and the Gehan–Breslow–Wilcoxon test. The former evaluates differences across the entire follow-up period, whereas the latter gives greater weight to earlier events. In some analyses, survival rates at specific time points, such as 3 and 5 years, were additionally compared using Z tests based on standard errors calculated from Greenwood’s formula. Using the 25% chronologically trimmed mean urinary pH, patients were stratified into two groups, urinary pH ≥7.0 and urinary pH <7.0, and OS was compared in the overall cohort and in subgroups defined by RAS wild-type vs RAS mutation-positive disease, liver metastasis present vs absent, and postoperative recurrence present vs absent.

Because these analyses were exploratory, no formal correction for multiple comparisons was applied, and p values were interpreted cautiously. All statistical analyses were performed using JMP Pro for Windows (version 13.0; SAS Institute Inc., Cary, NC, USA).

## 3 Results

### 3.1 Patient cohort and overall survival

**Figure 1:**
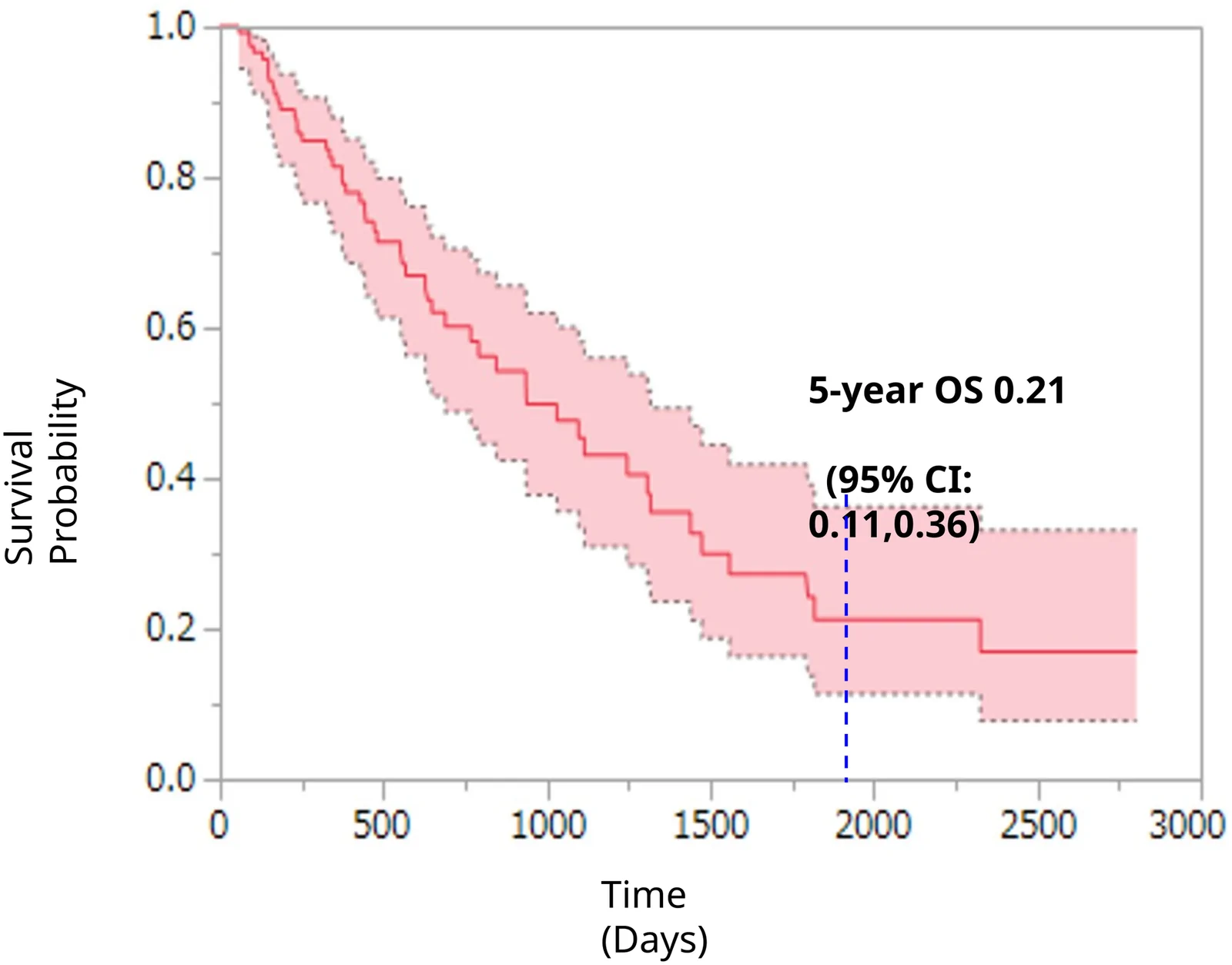
Overall survival of patients with stage IV or recurrent colorectal cancer treated with alkalization therapy (first visit 2014–2019; BRAF-mutant excluded; n = 129). Kaplan–Meier estimate of overall survival (OS) in patients with stage IV or recurrent colorectal cancer who first visited Karasuma Wada Clinic between 2014 and 2019 and received alkalization therapy in addition to standard-of-care systemic treatment (n = 129; BRAF-mutant cases excluded). Time zero was defined as the date of first visit. The shaded area indicates the 95% confidence interval. The 5-year OS rate was 0.212 (95% CI, 0.113–0.362).

A total of 129 patients with stage IV or recurrent colorectal cancer who first visited Karasuma Wada Clinic between 2014 and 2019 and underwent alkalization therapy were included in the primary analysis after exclusion of two patients with BRAF-mutant tumors. Patient characteristics are summarized in **Table 1**. The 5-year overall survival (OS) rate in this cohort was 0.212 (95% CI, 0.113–0.362), which appears favorable compared with the generally reported outcomes of stage IV colorectal cancer, despite the advanced disease status of the cohort (**Figure 1**).

**Table 1:** Patient characteristics of the primary analysis cohort (first visit 2014–2019; BRAF-mutant cases excluded; n = 129).

Characteristic	All patients (n = 129) (including unknown RAS status)	Known RAS status (n = 80)
Age (years), median [IQR]	64 [48–67]	64 [43–70]
Male: Female	46:83	26:54
RAS status wild-type: mutant (unknown)	37:43 (49)	37:43 (0)
Postoperative recurrence (+:−)	72:52 (unknown = 5)	47:33
Metastatic lesions		
Liver involvement		
No liver metastasis	47 (36%)	31 (39%)
Liver only	23 (18%)	12 (15%)
Liver + extrahepatic (multiple)	59 (46%)	37 (46%)
Metastatic sites (presence)		
Lung	68 (53%)	46 (58%)
Peritoneum	34 (26%)	24 (30%)
Bone	24 (19%)	16 (20%)

Data are presented as n (%) unless otherwise indicated. Continuous variables are shown as median [interquartile range]. The primary analysis cohort included patients with stage IV disease at initial presentation or postoperative recurrence after prior curative-intent surgery who underwent alkalization therapy at Karasuma Wada Clinic. Two BRAF-mutant cases were excluded. Liver metastasis was classified as absent, liver-only, or liver plus other organs. RAS status was categorized as wild-type, mutation-positive, or unknown. Abbreviations: BRAF, v-Raf murine sarcoma viral oncogene homolog B1; RAS, rat sarcoma viral oncogene homolog.

### 3.2 Exploratory analysis according to the components of alkalization therapy

In response to differences in the components of alkalization therapy, patients with known RAS status were classified into four groups: dietary intervention alone (n = 18), dietary intervention plus sodium bicarbonate (n = 35), dietary intervention plus citric acid (n = 4), and dietary intervention plus both sodium bicarbonate and citric acid (n = 23). The 25% chronologically trimmed mean urinary pH did not differ significantly among the four groups (ANOVA, p = 0.665), and no significant pairwise differences were observed by Tukey’s multiple comparison test. Similar results were observed when the analysis was performed separately in the RAS wild-type and RAS mutation-positive subgroups.

### 3.3 Primary Cox model with interaction terms

Among the 80 patients with known RAS mutation status (37 RAS wild-type and 43 RAS mutation-positive; all BRAF-mutant cases excluded), a multivariable Cox proportional hazards model was fitted using the 25% chronologically trimmed mean urinary pH as a continuous variable, together with RAS mutation status, liver metastasis, postoperative recurrence, sex, and age at first visit. Two interaction terms, urinary pH × RAS mutation status and urinary pH × liver metastasis, were simultaneously introduced into the model (**Table 2**). The interaction between urinary pH and RAS mutation status was significant (HR, 0.22; 95% CI, 0.06–0.78; likelihood ratio test, p = 0.0199), indicating that the prognostic impact of urinary pH was not uniform across molecular subgroups. In contrast, the interaction between urinary pH and liver metastasis was not significant (likelihood ratio test, p = 0.2456). These findings suggested that, in this cohort, the effect of urinary pH on prognosis was more appropriately interpreted as being modified by RAS status rather than as a single main effect.

**Table 2:** Multivariable Cox proportional hazards model for overall survival in patients with known RAS status, including interaction terms (n = 80).

Variable	Hazard ratio (HR)	95% CI	p value
Sex (female vs male)	1.614	0.674–3.868	0.2830
Age at diagnosis (per year)	1.002	0.969–1.037	0.8959
RAS mutation (yes vs no)	1.895	0.933–3.848	0.0770
Liver metastasis (yes vs no)	2.991	1.328–6.736	0.0082
Postoperative recurrence (no vs yes)	0.829	0.383–1.797	0.6349
Urinary pH (25% trimmed mean, continuous)	0.605	0.300–1.220	0.1600
RAS mutation × urinary pH (interaction)	0.215	0.059–0.784	0.0199
Liver metastasis × urinary pH (interaction)	0.421	0.098–1.812	0.2456

A multivariable Cox proportional hazards model was fitted in patients with known RAS status using the 25% chronologically trimmed mean urinary pH as a continuous variable. Covariates included urinary pH, RAS mutation status, liver metastasis, postoperative recurrence, sex, and age at first visit. Two interaction terms, urinary pH × RAS mutation status and urinary pH × liver metastasis, were simultaneously included in the model. Hazard ratios (HRs) with 95% confidence intervals (CIs) are shown. Because an interaction term was significant, the main effects of urinary pH and RAS mutation status should not be interpreted in isolation. CI, confidence interval; HR, hazard ratio; OS, overall survival; RAS, rat sarcoma viral oncogene homolog.

### 3.4 Survival according to RAS mutation status

**Figure 2:**
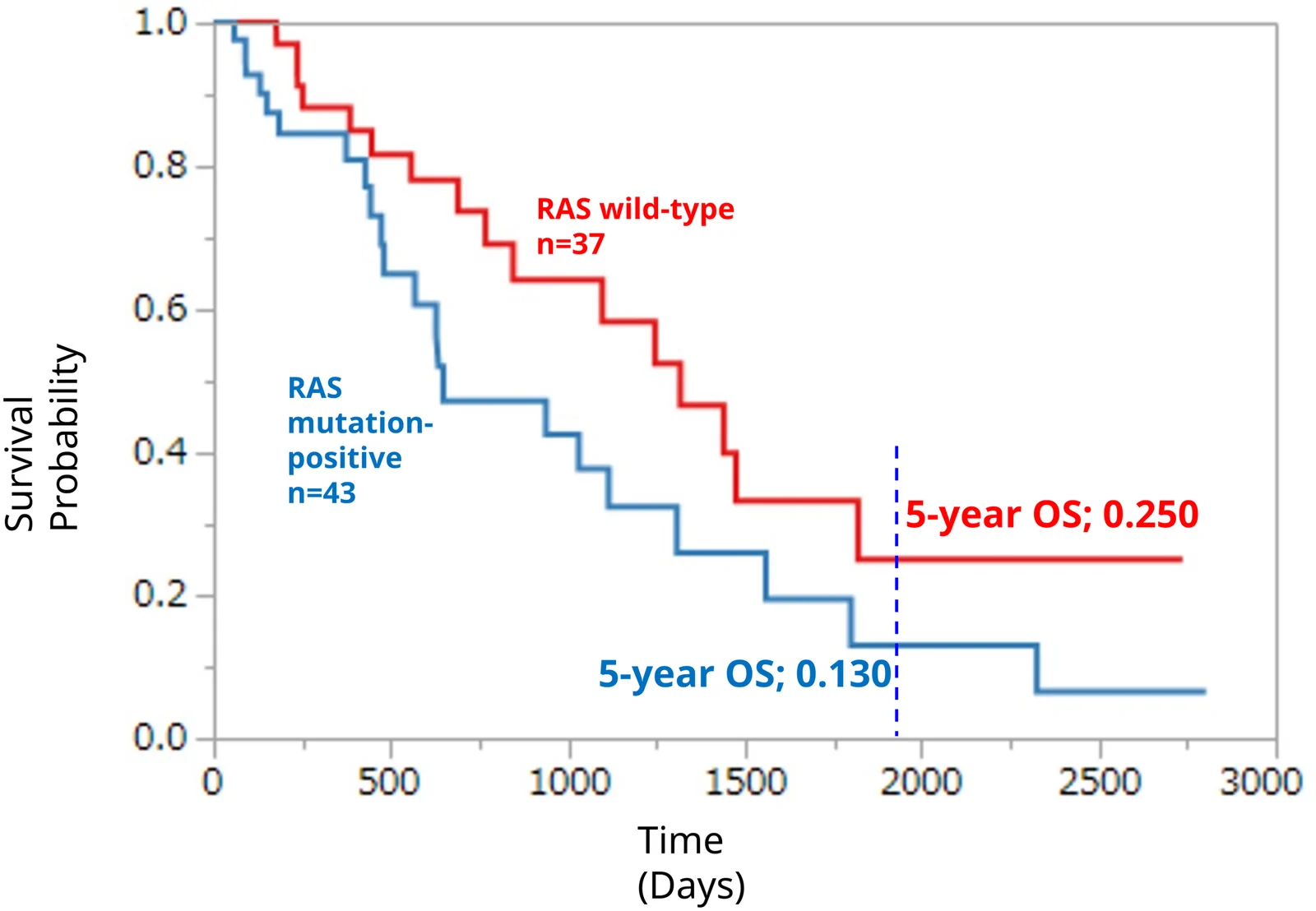
Overall survival stratified by RAS mutation status among patients with known RAS status (n = 80). Kaplan–Meier curves of overall survival (OS) stratified by RAS mutation status among patients with known RAS status (first visit 2014–2019; BRAF-mutant cases excluded; n = 80). The RAS wild-type group (n = 37) showed longer OS than the RAS mutation-positive group (n = 43). The 5-year OS rates were 0.250 and 0.130, respectively. Survival curves were compared using the log-rank test (two-sided p = 0.065).

Because the main effects of urinary pH and RAS mutation cannot be interpreted independently once a significant interaction is present, the association between urinary pH and OS was subsequently visualized by Kaplan–Meier analysis after stratification by RAS status. Among the 80 patients with known RAS status, the 5-year OS rates were 0.250 in the RAS wild-type group and 0.130 in the RAS mutation-positive group (**Figure 2**). Thus, patients with RAS wild-type tumors tended to have better survival than those with RAS mutation-positive tumors. The difference in survival curves was borderline significant by the two-sided log-rank test (p = 0.065), corresponding to a one-sided p value of approximately 0.032, and the 3-year survival rates at 1,095 days also differed significantly by Greenwood’s Z test (one-sided p = 0.028). Overall, these findings were consistent with the established view that RAS mutation-positive disease is associated with poorer prognosis.

### 3.5 Survival according to urinary pH in RAS-mutant disease

**Figure 3:**
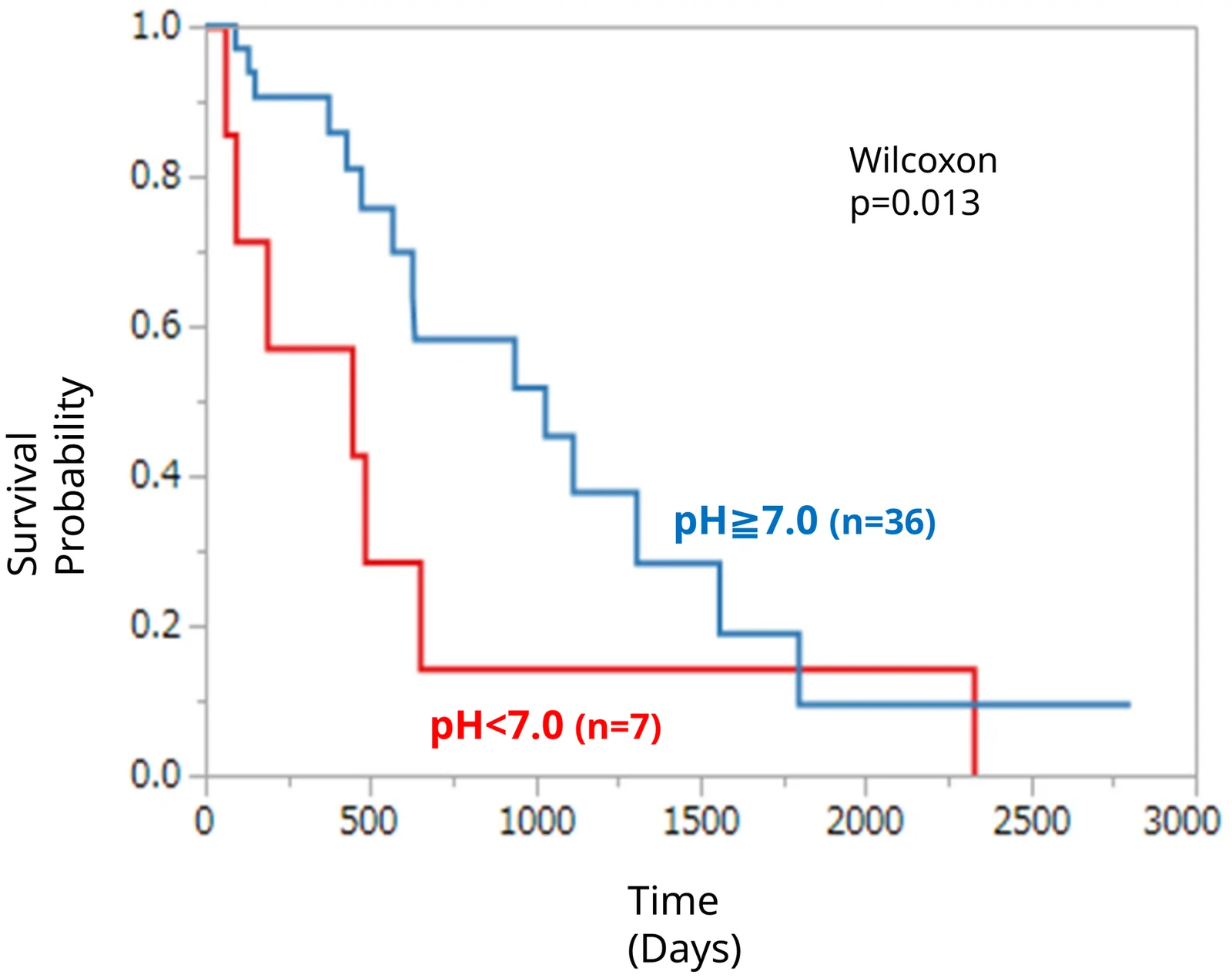
Overall survival according to urinary pH group in RAS mutation-positive patients (n = 43). Kaplan–Meier curves of overall survival (OS) stratified by urinary pH group in patients with RAS mutation-positive colorectal cancer (n = 43). Urinary pH was summarized using the 25% chronologically trimmed mean urinary pH, defined as the mean of the middle 50% of serial measurements after exclusion of the earliest and latest 25% in chronological order, and then dichotomized at 7.0 (≥7.0 vs <7.0). The urinary pH ≥7.0 group (n = 36) showed longer OS than the urinary pH <7.0 group (n = 7) (Gehan–Breslow–Wilcoxon test, two-sided p = 0.013).

When patients were stratified using the 25% chronologically trimmed mean urinary pH dichotomized at 7.0, a clear difference emerged within the RAS mutation-positive subgroup. In these 43 patients, those with urinary pH ≥7.0 (n = 36) showed a right-shifted survival curve compared with those with urinary pH <7.0 (n = 7), and the difference was significant by the Gehan–Breslow–Wilcoxon test (two-sided p = 0.013; **Figure 3**). A similar tendency was observed in the RAS wild-type subgroup, but the difference appeared smaller.

**Figure 4:**
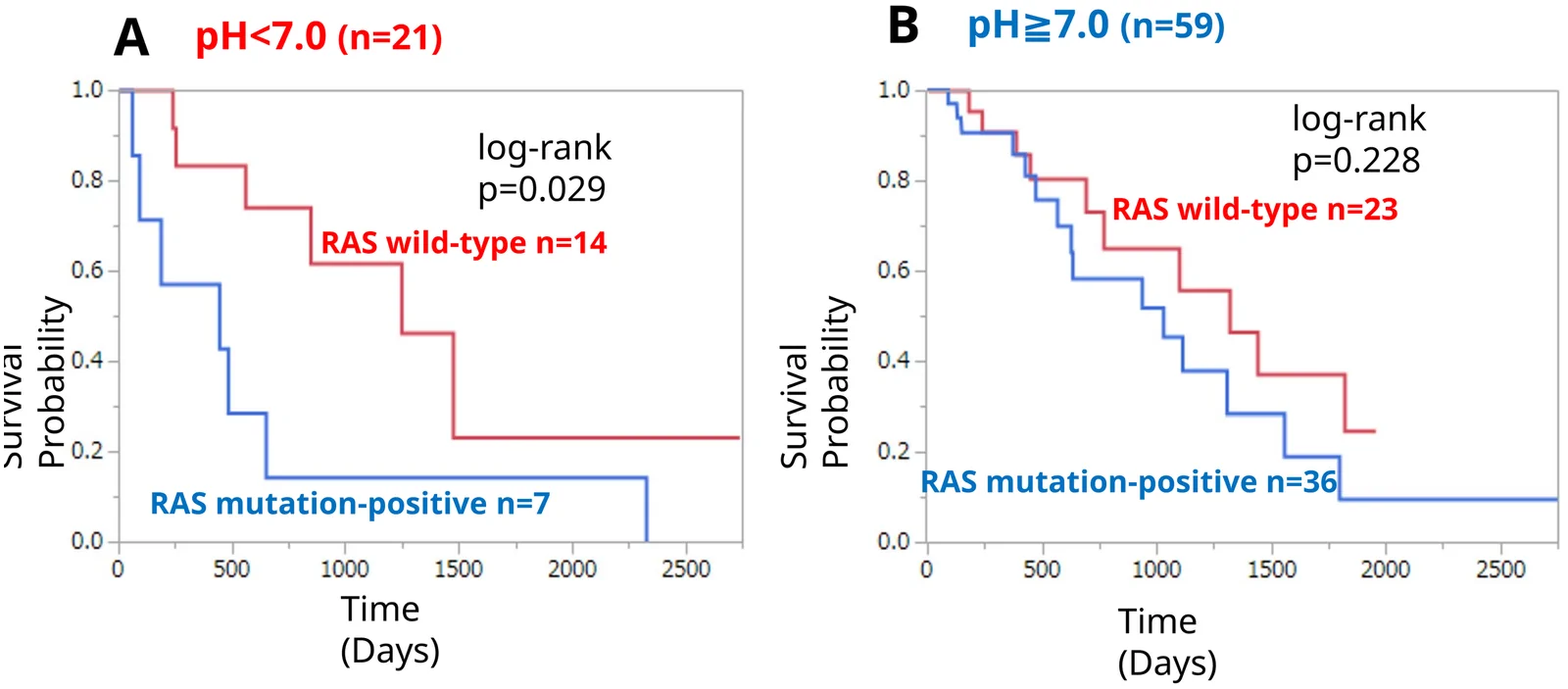
Comparison of overall survival between RAS wild-type and RAS mutation-positive patients within urinary pH strata. Kaplan–Meier curves comparing overall survival (OS) between RAS wild-type and RAS mutation-positive patients within urinary pH strata defined by the 25% chronologically trimmed mean urinary pH dichotomized at 7.0. **(A)** Urinary pH <7.0: OS was significantly better in RAS wild-type patients (n = 14) than in RAS mutation-positive patients (n = 7) (log-rank test, two-sided p = 0.029). **(B)** Urinary pH ≥7.0: No significant difference in OS was observed between RAS wild-type patients (n = 23) and RAS mutation-positive patients (n = 36) (log-rank test, two-sided p = 0.228).

To further examine the interaction between urinary pH and RAS status, OS was compared between RAS wild-type and RAS mutation-positive patients within each urinary pH stratum. In the urinary pH <7.0 stratum, survival was significantly better in the RAS wild-type group (n = 14) than in the RAS mutation-positive group (n = 7) (log-rank p = 0.029; **Figure 4A**). By contrast, in the urinary pH ≥7.0 stratum, the two survival curves became much closer, and no significant difference was observed between RAS wild-type (n = 23) and RAS mutation-positive patients (n = 36) (log-rank p = 0.228; **Figure 4B**). These findings suggest that, in patients who achieved sufficiently high urinary pH, the adverse prognostic impact of RAS mutation may have been attenuated.

### 3.6 Stratified analysis by liver metastasis

**Figure 5:**
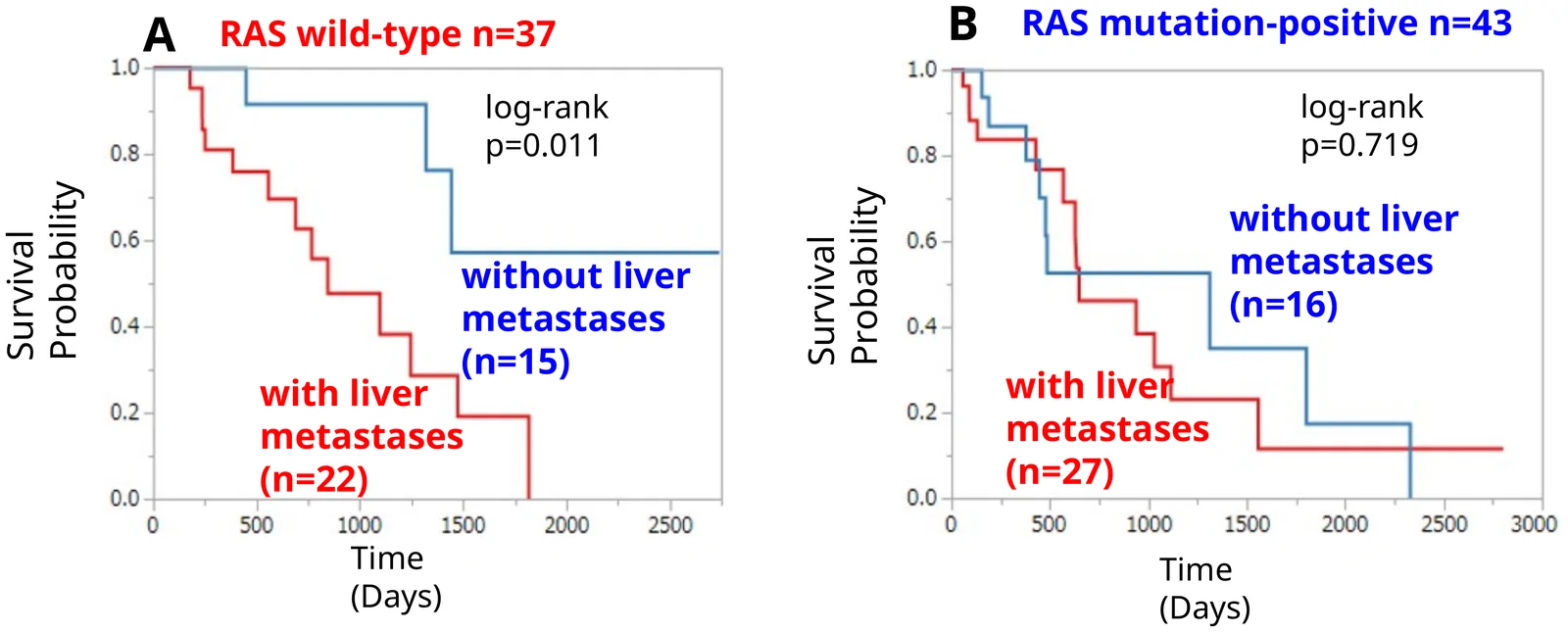
Overall survival according to liver metastasis status stratified by RAS status. **(A)** Kaplan–Meier curves of overall survival (OS) in RAS wild-type patients (n = 37), comparing those without liver metastases (n = 15) and those with liver metastases (n = 22) (log-rank p = 0.011). **(B)** Kaplan–Meier curves of OS in RAS mutation-positive patients (n = 43), comparing those without liver metastases (n = 16) and those with liver metastases (n = 27) (log-rank p = 0.719).

The effect of urinary pH was further examined according to liver metastasis status. In the RAS wild-type group (n = 37), patients without liver metastases (n = 15) had significantly better OS than those with liver metastases (n = 22) (log-rank p = 0.011; **Figure 5A**). In contrast, in the RAS mutation-positive group (n = 43), no clear difference in OS was observed between patients without liver metastases (n = 16) and those with liver metastases (n = 27) (log-rank p = 0.719; **Figure 5B**). Thus, the prognostic impact of liver metastasis appeared to differ according to RAS background.

**Figure 6:**
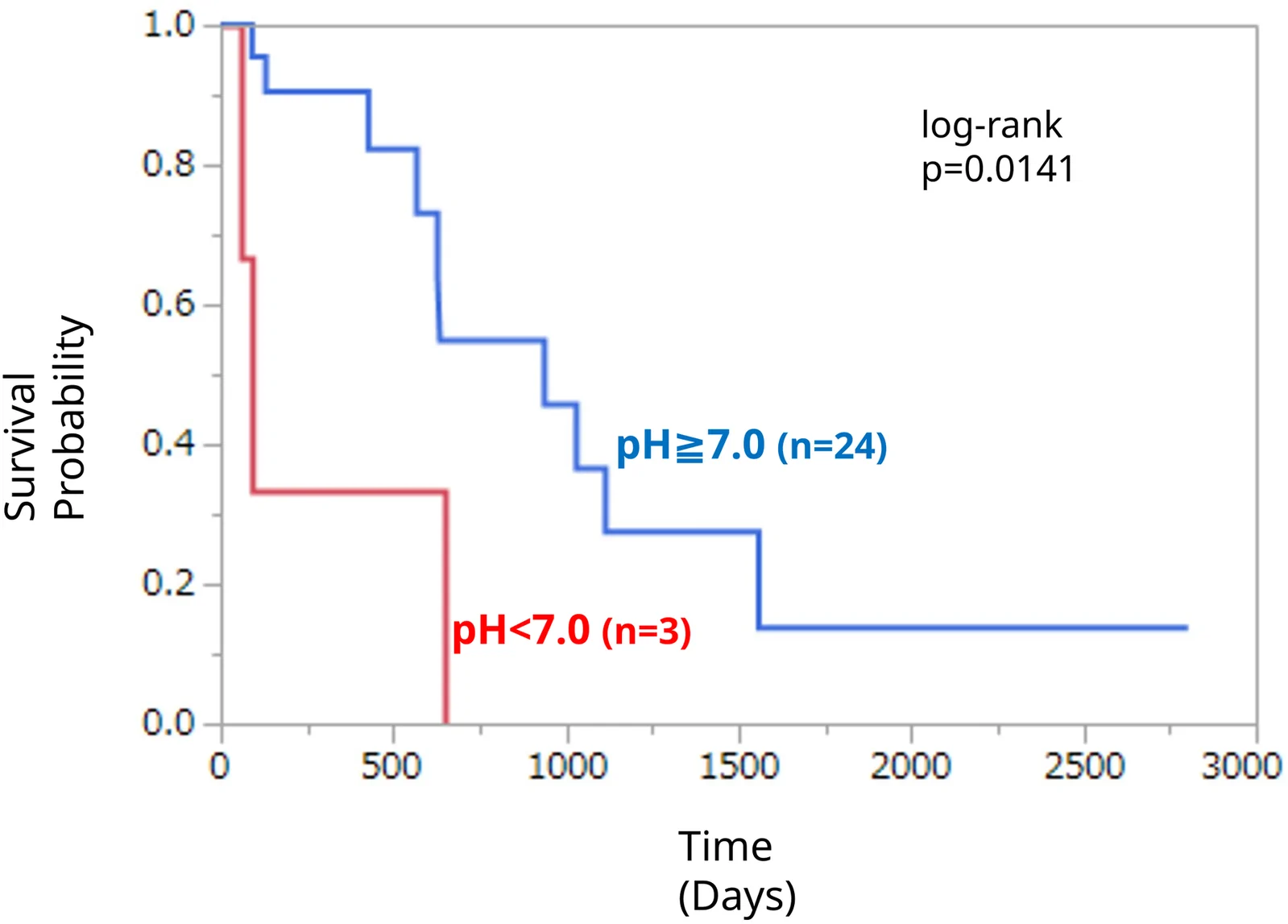
Overall survival according to urinary pH group in RAS mutation-positive patients with liver metastases (n = 27). Kaplan–Meier curves of OS stratified by urinary pH group in the subgroup of RAS mutation-positive patients with liver metastases. Urinary pH was dichotomized at 7.0 using the 25% chronologically trimmed mean urinary pH. The urinary pH ≥7.0 group (n = 24) showed longer OS than the urinary pH <7.0 group (n = 3) (log-rank p = 0.0141). Because the urinary pH <7.0 subgroup was very small, the result should be interpreted cautiously.

In the subgroup of patients with both RAS mutation-positive disease and liver metastases (n = 27), urinary pH remained strongly associated with OS. When dichotomized at 7.0 using the 25% chronologically trimmed mean urinary pH, patients with urinary pH ≥7.0 (n = 24) had significantly longer OS than those with urinary pH <7.0 (n = 3) (log-rank p = 0.0141; **Figure 6**). Therefore, even in this clinically high-risk subgroup characterized by both RAS mutation and liver metastasis, maintenance of urinary pH at or above 7.0 was associated with longer survival. However, because the urinary pH <7.0 group was extremely small, this result should be interpreted with caution.

### 3.7 Exploratory analysis of metastatic burden

We further examined whether urinary pH was associated with metastatic burden, defined by the number of metastatic organ sites. In both RAS wild-type and RAS mutation-positive patients, the 25% chronologically trimmed mean urinary pH did not differ significantly according to the number of metastatic sites. In addition, when patients were dichotomized by metastatic burden (<3 vs ≥3 metastatic sites), no significant difference in overall survival was observed in either RAS subgroup. These findings suggest that urinary pH was not simply a surrogate marker of the number of metastatic sites in this cohort. Rather, the prognostic association of urinary pH appeared to be more closely related to the interaction with RAS mutation status and liver metastasis status than to metastatic burden alone.

### 3.8 Survival according to postoperative recurrence status

Finally, among patients with available data on postoperative recurrence status, the 5-year OS rates were approximately 0.21 in the postoperative recurrence group (n = 72) and 0.22 in the *de novo* stage IV group (n = 52), indicating no major difference in long-term survival. Moreover, when postoperative recurrence status was examined separately within the RAS wild-type and RAS mutation-positive subgroups, no clear prognostic differences were identified. Taken together, these findings suggest that, once patients entered the clinical state of advanced or recurrent colorectal cancer and underwent alkalization therapy, prognosis may have been more strongly associated with the interaction between urinary pH and molecular background, particularly RAS status, than whether metastasis was present initially or developed after prior surgery.

## 4 Discussion

The most important finding of the present study is that, even among patients with RAS mutation-positive stage IV or recurrent colorectal cancer, in whom anti-EGFR antibodies such as cetuximab and panitumumab are not expected to be effective, patients who maintained urinary pH ≥ 7.0 under alkalization therapy showed substantially prolonged survival. Whereas patients with RAS wild-type tumors may benefit from anti-EGFR-targeted treatment, those with RAS-mutant tumors generally have fewer therapeutic options and a poorer prognosis (2–4). In this clinically unfavorable subgroup, the observation that the survival curve of patients who achieved sufficient alkalization approached that of the RAS wild-type group suggests that survival improvement may be achieved through a mechanism distinct from directly targeting oncogenic mutations themselves.

When stratified by liver metastasis status, Kaplan–Meier analysis showed that, among patients with RAS wild-type tumors, the presence of liver metastases was associated with significantly worse overall survival (OS) (log-rank p = 0.0109; **Figure 5A**). In contrast, no clear difference according to liver metastasis status was observed in the RAS mutation-positive group (p = 0.7193; **Figure 5B**). These results suggest that the prognostic impact of liver metastasis may differ depending on the molecular background defined by RAS status. Against this background, we further focused on the subgroup expected to have the poorest prognosis, namely patients with RAS mutation-positive disease and liver metastases, and examined the association between urinary pH and survival in this subgroup.

In that subgroup (n = 27), patients with urinary pH ≥ 7.0 (n = 24) had significantly better OS than those with urinary pH < 7.0 (n = 3) (log-rank p = 0.0141; **Figure 6**). Thus, even under the adverse condition of liver metastasis, which is usually regarded as a major poor prognostic factor, maintaining urinary pH at or above 7.0 was associated with longer survival. At the same time, the urinary pH < 7.0 group was very small, and therefore the precision of this estimate is limited. This finding should be interpreted cautiously and regarded as hypothesis-generating until validated in external datasets.

An additional strength of this study is that we did not rely solely on subgroup comparisons. Instead, we first used a Cox proportional hazards model including interaction terms and demonstrated that the association between urinary pH and OS was modified by RAS mutation status. Specifically, the interaction between urinary pH and RAS mutation was significant (HR 0.22, 95% CI 0.06–0.78; likelihood ratio test p = 0.0199), indicating that the prognostic impact of urinary pH was not uniform across molecular subgroups. We then visualized this structure using Kaplan–Meier curves and further showed that even in the high-risk subgroup of patients with both RAS mutation and liver metastasis, the urinary pH ≥ 7.0 group had significantly better survival. Taken together, these analyses support the clinical relevance of alkalization from multiple analytical perspectives.

Standard treatment for RAS mutation-positive metastatic colorectal cancer consists mainly of cytotoxic chemotherapy, such as FOLFOX- or FOLFIRI-based regimens, combined with anti-VEGF therapy, whereas anti-EGFR antibodies are not indicated. Accordingly, survival in patients with RAS-mutant disease is generally inferior to that in patients with RAS wild-type disease (2–4). In the present cohort, however, the survival curve of the RAS mutation-positive group shifted to the right when urinary pH was maintained at ≥ 7.0, and the gap in 5-year survival between RAS-mutant and RAS wild-type disease appeared to narrow. This observation is biologically plausible in light of increasing evidence that tumor acidity contributes to invasion, metastasis, therapeutic resistance, and immune suppression (5–16, 23).

The tumor microenvironment is often characterized by hypoxia, acidity, and high lactate concentrations, largely driven by the Warburg effect and altered proton transport. Such conditions are thought to contribute to poor outcomes through multiple mechanisms, including reduced intracellular uptake of anticancer drugs, impairment of drug activity at target sites, suppression of T-cell and NK-cell function, and compromised microcirculation at the level of small vessels (6, 13–18, 23). Alkalization therapy, consisting of a low-PRAL diet and oral alkalizing agents, may reduce the systemic acid load reflected in urinary pH and shift the metabolic and immune milieu toward a more physiological state. Importantly, urinary pH should not be interpreted as a direct surrogate for local extracellular tumor pH; rather, as emphasized in the recent narrative review by Suzuki, Kachi, and Wada, it should be regarded as a clinically available indirect supportive marker of systemic acid-base and metabolic conditions (23). Nevertheless, intervention at the level of the “field” or tumor–host environment may enhance the effectiveness of chemotherapy (with or without anti-EGFR therapy) and possibly support antitumor immune responses, thereby contributing to prolonged survival (17–23).

As an additional exploratory analysis, we reviewed serial urinary pH profiles in six patients who received anti-VEGF therapy at Karasuma Wada Clinic. Among these six patients, five subsequently received anti-EGFR therapy, whereas one received anti-VEGF therapy only.

In this small number of evaluable patients, no consistent upward or downward trend in urinary pH was observed before and after the initiation of anti-VEGF therapy. In the five patients who subsequently received anti-EGFR therapy, no consistent directional change in urinary pH was observed during the period of anti-EGFR therapy either. Because the number of patients was small and the timing of urinary pH measurements was heterogeneous, these observations should be interpreted cautiously as exploratory findings.

Importantly, the present retrospective study did not directly measure extracellular pH in the tumor microenvironment. Urinary pH was used as a clinically available marker reflecting dietary acid load, proton excretion, and systemic acid-base and metabolic conditions, but it cannot be regarded as a direct measurement of tumor acidity. Immunohistochemical evaluation of tumor acidity-related markers such as carbonic anhydrase IX (CA9) or LAMP2B, as suggested by the reviewer, would be valuable. However, because this was a retrospective observational study and tumor tissue specimens suitable for such analyses were not systematically available, these analyses could not be performed in the present study. Future prospective studies should incorporate tissue-based and/or imaging-based assessments of tumor acidity to clarify whether alkalization-oriented interventions alter the acidity of the tumor microenvironment itself.

The findings related to liver metastasis can also be interpreted within the framework of science-based medicine (SBM). The liver is a major metastatic niche favored by glycolysis-dominant tumor cells and provides abundant blood flow, glucose availability, oxygen gradients, and an immunotolerant microenvironment. Therefore, the overall adverse impact of liver metastasis in our cohort is consistent with previous large cohort studies (2, 3, 25). However, after the introduction of alkalization therapy, the difference between patients with and without liver metastases appeared relatively modest, particularly among those with RAS-mutant tumors who achieved urinary pH ≥ 7.0. Although this finding must be interpreted cautiously because of the limited sample size, it raises the possibility that reducing systemic acid load and partially correcting the acidic milieu, including that of the liver, may attenuate the adverse impact of liver metastasis. From an SBM perspective, this is consistent with the view of cancer as a non-equilibrium open system whose behavior is shaped not only by tumor genetics but also by the host environment (26–30).

Another noteworthy finding was the absence of a major survival difference between patients who presented with stage IV disease at the first visit and those who developed postoperative recurrence. In general, postoperative recurrent disease is often assumed to represent a somewhat more indolent biological course than *de novo* stage IV disease. In the present cohort, however, the 5-year survival rates were nearly identical. This suggests that, once patients entered the clinical state of advanced or recurrent colorectal cancer and underwent alkalization therapy, prognosis may have been influenced more strongly by molecular background (RAS mutation status) and by the degree of alkalization achieved, rather than by whether the disease was present at initial diagnosis or developed after prior surgery.

Published large-scale datasets indicate that median survival in stage IV colorectal cancer generally remains in the range of approximately 2–3 years and that the 5-year survival rate is about 10–15% (2, 3, 25). In contrast, the present cohort, despite consisting entirely of patients with stage IV disease or recurrent colorectal cancer, achieved an overall 5-year survival rate of approximately 21%. Moreover, as previously observed in our analyses of lung and pancreatic cancer, patients who maintained urinary pH ≥ 7.0 showed a characteristic long-tail survival pattern. Such a phenomenon cannot be fully explained by a modest shift in median survival alone and may instead reflect a subgroup of patients in whom the tumor–host system transitioned toward a more controlled and stable biological state (19–23).

From the perspective of complex systems and systems biology, both living organisms and tumors can be regarded as non-equilibrium open systems that maintain order through exchanges of matter and energy with the environment (26–30). In this context, an acidic tumor microenvironment may be viewed as a self-sustaining malignant regime, in which glycolysis dominance, hypoxia, proton export, and immune suppression reinforce one another. Buffering strategies and dietary interventions may act as levers that weaken this coupling. The long-tail survival observed among patients with urinary pH ≥ 7.0 in the present study may therefore represent a hypothesis-generating signal that intervention and host adaptation shifted the tumor–host system toward a different and more favorable attractor state.

An important methodological message of this study is that the key issue was not simply whether urinary pH had a statistically significant main effect on OS, but whether its prognostic effect was modified by RAS mutation status. The discrepancy between the Kaplan–Meier analyses, which suggested a survival difference by urinary pH group, and multivariable Cox models, in which urinary pH did not always emerge as an independent factor, is consistent with a setting in which the effect of pH is not uniform across all patients. Incorporating the urinary pH × RAS interaction term allowed this apparent discrepancy to be interpreted in a more statistically coherent manner.

This study has several important limitations. First, it was a single-center retrospective observational study, and selection bias as well as treatment allocation bias cannot be fully excluded. Second, the implementation and adherence to alkalization therapy were not uniform across patients, and urinary pH is both an index of intervention and a reflection of lifestyle and general condition; therefore, causal direction cannot be established from the present data alone. Third, the sample size was limited relative to large clinical trials, particularly in subgroup analyses such as RAS mutation-positive patients with urinary pH < 7.0, and the interaction analysis should therefore be regarded as exploratory. In addition, although proportional hazards assumptions were checked and the number of covariates was chosen with attention to event counts to reduce overfitting, the possibility of model instability remains. These findings should therefore be interpreted as a strong hypothesis-generating signal requiring validation in independent cohorts and prospective studies.

Despite these limitations, the present results support the hypothesis that even in RAS mutation-positive stage IV or recurrent colorectal cancer, where certain molecularly targeted agents are ineffective, intervention directed at metabolism and the tumor microenvironment through alkalization therapy may improve at least part of the medium- to long-term prognosis. Taken together with our previous reports in lung and pancreatic cancer, these findings suggest that controlling the acidity of the tumor microenvironment may represent a novel therapeutic axis that is partly independent of conventional TNM staging and driver mutation status.

## 5 Summary and future perspectives

Our findings strongly support the working hypothesis that, even in RAS mutation-positive colorectal cancer, where effective targeted treatment options are limited, survival may be meaningfully prolonged by improving the tumor microenvironmental “field” through alkalization. Stage IV colorectal cancer remains a highly difficult disease, with reported 5-year survival rates generally around 10–15% (2, 3, 25). In this context, the observation that patients who maintained urinary pH ≥ 7.0 showed both improved 5-year survival and long-tail survival is an important signal from the perspective of SBM, which focuses on the tumor–host environment, especially systemic acid-base balance. The fact that favorable outcomes associated with urinary pH ≥ 7.0 were observed across strata defined by liver metastasis and postoperative recurrence further suggests that alkalization therapy may function at the level of a systemic environmental determinant rather than merely a local factor (19–23, 26–30).

At the same time, the present study has multiple constraints, including its retrospective single-center design, the lack of a non-alkalization control group, the limited availability of detailed metabolic markers beyond urinary pH, and the small size of key subgroups, especially RAS mutation-positive patients with urinary pH < 7.0 and those with liver metastases. Differences in general condition, nutritional status, and concomitant systemic treatments may also have acted as confounders. Therefore, it is not possible to conclude definitively from the present data that alkalization itself improved prognosis.

Nevertheless, the observation that a simple and non-invasive marker—higher urinary pH—was consistently associated with better outcomes across strong conventional prognostic factors such as RAS/BRAF mutation status, liver metastasis, and postoperative recurrence provides a compelling signal for future investigation. Prospective intervention studies evaluating standard treatment with or without alkalization-oriented support will be necessary to test this hypothesis rigorously.

Taken together, these findings suggest that alkalization-oriented treatment may become a cross-cutting strategy against the shared pathophysiological feature of microenvironmental acidification across multiple tumor types, including colorectal cancer in addition to lung and pancreatic cancer. The present study should therefore be regarded as an observational starting point for that broader research program.

## Data Availability

The data analyzed in this study are not publicly available due to privacy and ethical restrictions. Requests to access the datasets should be directed to the corresponding author and will be considered in accordance with institutional and ethical requirements.
